# Type-3 fuzzy logic and Lyapunov approach for dynamic modeling and analysis of financial markets

**DOI:** 10.1016/j.heliyon.2024.e33730

**Published:** 2024-07-01

**Authors:** Shu-Rong Yan, Ardashir Mohammadzadeh, Ebrahim Ghaderpour

**Affiliations:** a School of Finance, Guangzhou Huashang University, Guangzhou, 511300, China; b Department of Computational and Data Science, Astana IT University, Prospekt Mangilik Yel., Astana, 020000, Kazakhstan; c Earth and Space Inc., Calgary, T3A 5B1, Canada; d Department of Earth Sciences, Sapienza University of Rome, P.le Aldo Moro, 5, Rome, RM, 00185, Italy

**Keywords:** Fuzzy logic systems, Learning, Financial chaotic systems, Type-3 fuzzy logic, Lyapunov views, Financial markets

## Abstract

Chaos theory offers a new way to investigate variations in financial markets data that cannot be obtained with traditional methods. The primary approach for diagnosing chaos is the existence of positive small Lyapunov views. The positive Lyapunov index indicates the average instability and the system's chaotic nature. The negativity indicates the average rate of non-chaoticness. In this paper, a new approach on basis of type-3 fuzzy logic systems is introduced for modeling the chaotic dynamics of financial data. Also, the attracting dimension tests and the Lyapunov views in the reconstructed dynamics are used for examinations. The simulations on case-study currency market show the applicability and good accuracy of the suggested approach.

## Highlights

• A novel type-3 fuzzy logic system based model is proposed for financial chaotic systems.
• The results on Logistics and Henon systems showed good modeling accuracy.
• The model was applied to time series of exchange rate parity in the financial markets.
• The suggested fuzzy based approach showed excellent accuracy in modeling.

## 1 Introduction

The theory of chaotic systems (CSs) is a novel way to model economics, especially in financial markets. Chaotic systems are observed in nature as well as in human behavior. For instance, heartbeats, clock pendulums, and economic fluctuations all exhibit nonlinear dynamic behavior. According to this theory, events in the world are so complex and dynamic that they seem chaotic, but in fact, the chaotic system has an underlying order. It is challenging to identify this hidden order, although it is not impossible because several factors in dynamic and unpredictable interaction shape the behavior of phenomena and create their future behavior pattern. Research on the chaotic existence in economic problems has been started since 1980s. For example, Stutzer [1] introduced a macroeconomic growth model with chaotic dynamics. Barnett and Chen [2] showed that financial factors show chaotic behavior. Shintani and Linton [3] have also explored the existence of chaos in various economic indicators. The presence of chaos in the exchange rate of Romania against the US dollar was investigated by Scarlat et al. [4]. Using the BDS test and the Lyapunov view, and correlation dimension, existence of chaos in both periods was confirmed. Lee-Chua [5] studied the existence of chaos on the Philippine peso against the US dollar and used correlation dimension tests and the most extensive Lyapunov view. These tests are shown the presence of chaos [6].

Chaotic modeling and dynamic modeling of financial systems has been well studied [7], [8], [9]. For example, Diouf and Sene [10] used the Caputo derivative to construct a fractional-order model for financial systems, and bifurcation diagrams analyze factors, such as saving rate. Liping et al. [11] developed a chaotic model for financial systems using the Atangana-Baleanu operator and analyzed the chaotic behavior by the Lyapunov approach. Atangana et al. [12] developed the Mittag-Leffler technique to design a chaotic model and suggested an optimal controller based on the Euler-Lagrange scheme. Farman et al. [13] analyzed the minimum interest rate by a chaotic model and studied the stability range. Xu et al. [14] studied a chaotic model and analyzed the bifurcation phenomena and stability by designing a simple controller. Ma and Li [15] developed a three-dimensional (3D) model and introduced some conditions to stabilize an expanded model. Lu [16] proposed an intermittent controller for a chaotic financial model and analyzed instability in various conditions. Anh and Inoue [17] presented a dynamic model and compared it with Black-Scholes model.

The similarity of the time series of exchange rate parity in financial markets with chaotic time series and their random characteristics make possible the existence of some kind of chaotic nonlinear dynamics that can be discovered in these series. Every country in the international economic system interacts with other economies through two channels: one is the trade of goods and services and the other is the inflow and outflow of capital. As a result of this interaction, the country's domestic currency has a price in the global economy, called the exchange rate in the conventional economy. In purchasing power parity, the exchange rate shows the ratio of the prices of domestic goods in terms of domestic currency to the price of foreign goods in local currency.

Herein, the cases where the exchange rate forecasting and how its change process should be considered as a necessary matter in decision-making are briefly mentioned.

A- Speculation: A speculator enters into transactions with the risk of currency fluctuations in the currency market to make a profit. If changes prediction accuracy is good in the cash rates of the currency well, they will profit.

B- Uncovered Interest Arbitrage (IA): IA is related to the global flow of cash capital in the short term in order to earn more money from abroad. For this purpose, in order to avoid the loss of foreign currency devaluation, it is necessary to know how the exchange rate changes.

C- Foreign direct investment: In the evaluation of projects related to foreign direct investment, calculated by indicators such as Net Present Value (NPV) and Internal Rate of Return (IRR), in order to convert the cash flow of foreign money into domestic money, one needs to forecast the exchange rate. Because a plan that is acceptable in terms of foreign currency may not be acceptable in terms of domestic currency.

A leading way to find an uncertain system in economics is to verify that data exhibit a chaotic behavior. This idea is the hidden framework of a chaotic economy. Hence, there are two main groups of data perturbation tests: the first method is to look at the trajectory of the data when the initial conditions of the system are slightly changed [18]. By approximating the Lyapunov expression, this may be accomplished. The divergence of the mean route of experimental data produced by the system with an incredibly slight change in the beginning circumstances is quantified by the Lyapunov exponent. When there is a small change in the deterministic system, if the data change exponentially, then the Lyapunov exponent will be positive and therefore chaotic. The Lyapunov expression will be negative in the event that the convergence route resumes its steady state [19]. The next test for the existence of chaos is 2-system absorber dimension test. Unfortunately, the analysis becomes complicated in the presence of larger dimensions, and the test for chaos is weaker. This issue is a strong barrier to the theory of chaotic economics, and it is one of the main reasons that statistical research has not been aware of the existence of chaotic dynamics in the data [20].

To deal with uncertain, complicated systems, recently, type-3 fuzzy logic systems (T3-FLSs) have been suggested. T3-FLSs have been used for different applications. For example, Liu et al. [21] studied an energy management system and utilized a T3-FLS to handle the uncertainty of solar energy. Cao et al. [22] developed an energy modeling and forecasting technique is developed using T3-FLSs and verified the applicability of T3-FLSs. Vafaie et al. [23] established a control system by T3-FLSs and used to control a gyroscope with uncertain dynamics. Wang et al. [24] designed a fault detection system in Gas sensors and examined the better accuracy of T3-FLSs. Castillo et al. [25] developed a T3-FLS for image processing and suggested an approach to improve the image quality in televisions. Tian et al. [26] improved the dynamics of a case of financial systems using T3-FLSs. Xu et al. [27] designed an optimal controller based on T3-FLSs and analyzed the behavior of controlled system. Castillo et al. [28] studied a financial prediction problem and suggested a T3-FLS based scheme for better predication under chaotic and noisy data. Tarafdar et al. [29] developed an economic model using T3-FLSs and presented some statistical data to demonstrate the better outcome of T3-FLSs. Tian et al. [30] developed another T3-FLS based controller for chaotic systems and studied stability in the presence of estimation error of T3-FLSs.

In this paper, the chaotic modeling of financial systems is studied, then the basic approaches are analyzed, and finally a T3-FLS-based model is proposed and validated through synthetic and experimental data. The rest of this paper is organized as follows. In Section 2, the proposed T3-FLS model is presented. Section 3 investigates the concept of chaos and basic chaotic models, such as Henon and the logistic model. In Section 4, the attractor dimension tests are investigated. In Section 5 the simulations are given, and finally in Section 6 the conclusions are presented.

## 2 Type-3 FLS

**Figure 1:**
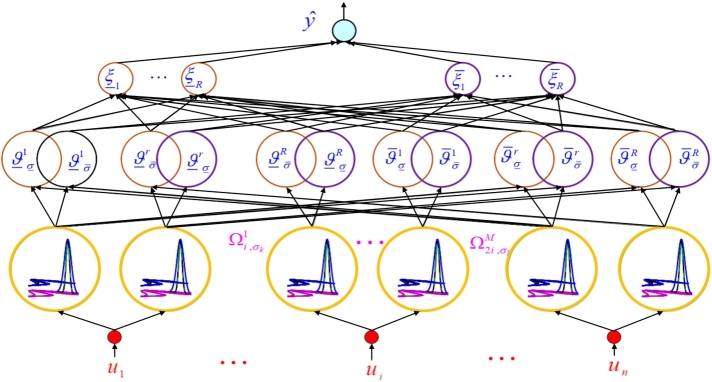
Structure of T3-FLS.

The chaotic dynamics are modeled by the suggested T3-FLS, and the identified model can be used for various applications. T3-FLSs are intricate and complex structures that are used to handle uncertainties in information. These systems have a higher level of complexity than type-1 and type-2 fuzzy logic systems, with additional degrees of freedom to represent the level of assigned uncertainty in the input data [31], [32]. In a T3-FLS, each linguistic term has a fuzzy set associated with it, which contains a spectrum of membership grades representing the degree to which that term might apply to the input data. These membership grades are then subjected to further fuzzification through additional mapping functions, resulting in a highly nuanced and detailed model of the input information. T3-FLSs are best suited for situations where the information available is highly uncertain or incomplete, and where a higher level of granularity and detail is required in the output for better decision-making. The T3-FLS diagram is given in Fig. 1. The structure shows the four layers. First, the inputs are received, and the memberships are computed. In the next layer, the firing degree of rules is calculated and finally the output is obtained. The details of all steps are presented below.

1) The inputs are the output signal in previous times, $u_{1}(t)=y(t-1)$, $u_{2}(t)=y(t-1)$ and $u_{n}(t)=y(t-n)$, where *n* is the number of inputs.

2) For $u_{1}(t)$, $u_{2}(t)$ and $u_{n}(t)$, *M* membership functions (MF) are considered. MFs show the changing range of inputs. For all MFs, the upper/lower memberships should be computed as:

$${\overset{¯}{\vartheta }}_{\Psi_{i,\;{\overset{¯}{\sigma }}_{k}}^{j}}\left(u_{i}\right)=\exp\left(-\frac{{\left(u_{i}-m_{\Psi_{i,\;{\overset{¯}{\sigma }}_{k}}^{j}}\right)}^{2}}{{\overset{¯}{\chi }}_{\Psi_{i,\;{\overset{¯}{\sigma }}_{k}}^{j}}^{2}}\right),$$

$${\overset{¯}{\vartheta }}_{\Psi_{i,\;{\underline{\sigma }}_{k}}^{j}}\left(u_{i}\right)=\exp\left(-\frac{{\left(u_{i}-m_{\Psi_{i,\;{\underline{\sigma }}_{k}}^{j}}\right)}^{2}}{{\overset{¯}{\chi }}_{\Psi_{i,\;{\underline{\sigma }}_{k}}^{j}}^{2}}\right),$$

$${\underline{\vartheta }}_{\Psi_{i,\;{\overset{¯}{\sigma }}_{k}}^{j}}\left(u_{i}\right)=\exp\left(-\frac{{\left(u_{i}-m_{\Psi_{i,\;{\overset{¯}{\sigma }}_{k}}^{j}}\right)}^{2}}{{\underline{\chi }}_{\Psi_{i,\;{\overset{¯}{\sigma }}_{k}}^{j}}^{2}}\right),$$

$${\underline{\vartheta }}_{\Psi_{i,\;{\underline{\sigma }}_{k}}^{j}}\left(u_{i}\right)=\exp\left(-\frac{{\left(u_{i}-m_{\Psi_{i,\;{\underline{\sigma }}_{k}}^{j}}\right)}^{2}}{{\underline{\chi }}_{\Psi_{i,\;{\underline{\sigma }}_{k}}^{j}}^{2}}\right),$$

where ${\overset{¯}{\vartheta }}_{\Psi_{i,{\overset{¯}{\sigma }}_{k}}^{j}}\left(u_{i}\right)$ and ${\overset{¯}{\vartheta }}_{\Psi_{i,\;{\underline{\sigma }}_{k}}^{j}}\left(u_{i}\right)$ are the upper memberships, and ${\underline{\vartheta }}_{\Psi_{i,\;{\overset{¯}{\sigma }}_{k}}^{j}}\left(u_{i}\right)$ and ${\underline{\vartheta }}_{\Psi_{i,\;{\underline{\sigma }}_{k}}^{j}}\left(u_{i}\right)$ represent the lower memberships.

3) The *r*-th rule is given as:

$$\begin{array}{c} \mathrm{Rule}\;\#r:\;\mathrm{If}\;u_{1}\;\mathrm{is}\;\;\Psi_{1,\;{\overset{¯}{\sigma }}_{k}}^{r}\;\mathrm{and}\;u_{2}\;\mathrm{is}\;\;\;\Psi_{2,\;{\overset{¯}{\sigma }}_{k}}^{r}\;\mathrm{and}\;\;u_{3}\;\;\mathrm{is}\;\;\Psi_{3,\;{\overset{¯}{\sigma }}_{k}}^{r},\;\;\;\;\mathrm{then}\;\;\overset{ˆ}{y}\in \left[{\underline{x}}_{r},{\overset{¯}{x}}_{r}\right], \end{array}$$

where $\Psi_{1,\;{\overset{¯}{\sigma }}_{k}}^{r}$, $\Psi_{2,\;{\overset{¯}{\sigma }}_{k}}^{r}$ and $\Psi_{3,\;{\overset{¯}{\sigma }}_{k}}^{r}$ are the *r*-th MF for $u_{1}$, $u_{2}$ and $u_{3}$, respectively. Rule firings are:

$${\underline{\xi }}_{{\overset{¯}{\sigma }}_{k}}^{r}={\underline{\vartheta }}_{\Psi_{1,\;{\overset{¯}{\sigma }}_{k}}^{j1}}\left(u_{1}\right){\underline{\vartheta }}_{\Psi_{2,\;{\overset{¯}{\sigma }}_{k}}^{j2}}\left(u_{2}\right){\underline{\vartheta }}_{\Psi_{3,\;{\overset{¯}{\sigma }}_{k}}^{j_{3}}}\left(u_{3}\right),$$

$${\underline{\xi }}_{{\underline{\sigma }}_{k}}^{r}={\underline{\vartheta }}_{\Psi_{1,\;{\underline{\sigma }}_{k}}^{j1}}\left(u_{1}\right){\underline{\vartheta }}_{\Psi_{2,\;{\underline{\sigma }}_{k}}^{j2}}\left(u_{2}\right){\underline{\vartheta }}_{\Psi_{3,\;{\underline{\sigma }}_{k}}^{j_{3}}}\left(u_{3}\right),$$

$${\overset{¯}{\xi }}_{{\overset{¯}{\sigma }}_{k}}^{r}={\overset{¯}{\vartheta }}_{\Psi_{1,\;{\overset{¯}{\sigma }}_{k}}^{j1}}\left(u_{1}\right){\overset{¯}{\vartheta }}_{\Psi_{2,\;{\overset{¯}{\sigma }}_{k}}^{j2}}\left(u_{2}\right){\overset{¯}{\vartheta }}_{\Psi_{3,\;{\overset{¯}{\sigma }}_{k}}^{j_{3}}}\left(u_{3}\right),$$

$${\overset{¯}{\xi }}_{{\underline{\sigma }}_{k}}^{r}={\overset{¯}{\vartheta }}_{\Psi_{1,\;{\underline{\sigma }}_{k}}^{j1}}\left(u_{1}\right){\overset{¯}{\vartheta }}_{\Psi_{2,\;{\underline{\sigma }}_{k}}^{j2}}\left(u_{2}\right){\overset{¯}{\vartheta }}_{\Psi_{3,\;{\underline{\sigma }}_{k}}^{j_{3}}}\left(u_{3}\right),$$

where ${\overset{¯}{\xi }}_{{\overset{¯}{\sigma }}_{k}}^{r}$ and ${\overset{¯}{\xi }}_{{\underline{\sigma }}_{k}}^{r}$ represent the upper firing degrees, and ${\underline{\xi }}_{{\overset{¯}{\sigma }}_{k}}^{r}$ and ${\underline{\xi }}_{{\underline{\sigma }}_{k}}^{r}$ denote the lower firing degrees.

4) The output $\overset{ˆ}{y}$ is obtained as [33]:

$$\begin{array}{c} \overset{ˆ}{y}=x^{T}\mu , \end{array}$$

where *x* and *μ* are:

$$x={\left[{\underline{x}}_{1},...,{\underline{x}}_{M},{\overset{¯}{x}}_{1},...,{\overset{¯}{x}}_{M}\right]}^{T},$$

$$\mu ={\left[{\underline{\mu }}_{1},...,{\underline{\mu }}_{M},{\overset{¯}{\mu }}_{1},...,{\overset{¯}{\mu }}_{M}\right]}^{T},$$

where *M* denote rule numbers, and ${\underline{\mu }}_{r}$ and ${\overset{¯}{\mu }}_{r}$ are:

$$\begin{array}{c} {\overset{¯}{\mu }}_{r}=\frac{\sum_{k=1}^{n_{\sigma }}{\overset{¯}{\sigma }}_{k}\frac{{\overset{¯}{\xi }}_{{\overset{¯}{\sigma }}_{k}}^{r}}{\sum_{r=1}^{M}\left({\overset{¯}{\xi }}_{{\overset{¯}{\sigma }}_{k}}^{r}+{\underline{\xi }}_{{\overset{¯}{\sigma }}_{k}}^{r}\right)}}{\sum_{k=1}^{n_{\sigma }}\left({\overset{¯}{\sigma }}_{k}+{\underline{\sigma }}_{k}\right)}+\frac{\sum_{j=1}^{n_{\sigma }}{\underline{\sigma }}_{k}\frac{{\overset{¯}{\xi }}_{{\underline{\sigma }}_{k}}^{r}}{\sum_{r=1}^{M}\left({\overset{¯}{\xi }}_{{\underline{\sigma }}_{k}}^{r}+{\underline{\xi }}_{{\underline{\sigma }}_{k}}^{r}\right)}}{\sum_{k=1}^{n_{\sigma }}\left({\overset{¯}{\sigma }}_{k}+{\underline{\sigma }}_{k}\right)},\;\;\;r=1,...,M, \end{array}$$

$$\begin{array}{c} {\underline{\mu }}_{r}=\frac{\sum_{k=1}^{n_{\sigma }}{\overset{¯}{\sigma }}_{k}\frac{{\underline{\xi }}_{{\overset{¯}{\sigma }}_{k}}^{r}}{\sum_{r=1}^{M}\left({\overset{¯}{\xi }}_{{\overset{¯}{\sigma }}_{k}}^{r}+{\underline{\xi }}_{{\overset{¯}{\sigma }}_{k}}^{r}\right)}}{\sum_{k=1}^{n_{\sigma }}\left({\overset{¯}{\sigma }}_{k}+{\underline{\sigma }}_{k}\right)}+\frac{\sum_{j=1}^{n_{\sigma }}{\underline{\sigma }}_{k}\frac{{\underline{\xi }}_{{\underline{\sigma }}_{k}}^{r}}{\sum_{r=1}^{M}\left({\overset{¯}{\xi }}_{{\underline{\sigma }}_{k}}^{r}+{\underline{\xi }}_{{\underline{\sigma }}_{k}}^{r}\right)}}{\sum_{k=1}^{n_{\sigma }}\left({\overset{¯}{\sigma }}_{k}+{\underline{\sigma }}_{k}\right)},\;\;\;r=1,...,M, \end{array}$$

where $n_{\sigma }$ denotes the number of slices. The rules are updated as:

x(t+1)=x(t)+γμe,

where *γ* is constant, and *e* denote the error between target and of output of T3-FLS.

## 3 Chaos

The traditional view of economic phenomena tries to model data linearly with an approach that has random processes. The disturbances and irregularities observed in them are caused by the random effect of multiple inputs and external shocks. In the 1960s and 1970s, it became clear that many seemingly complex and random natural processes can be modeled with less complex nonlinear equations with limited degrees of freedom. Such certain systems that show apparently random and unpredictable behavior are the basis for the introduction of chaos theory and the development of chaotic time series modeling. In the investigation of chaos, the reason for data fluctuations such as oil price data is the internal mechanism of the system that generates it, and external and random shocks have not led to the creation of such seemingly disorderly behaviors. Most of the natural phenomena that have completely non-linear behavior can be well studied and analyzed in chaos research. Although most of these macroeconomic phenomena have countless influential factors from the point of view of economists, they are modeled with a certain chaotic system with a limited dimension.

Chaotic systems are also sensitive to the circumstances at the beginning. Because the prediction error at each stage can be considered an error in the conditions. Assuming the beginning of the forecast in the next moments, the accuracy of the multi-stage forecast is exceptionally high. It is affected by the error in the previous steps and drops quickly. The properties of chaotic systems are investigated with two essential concepts: absorber dimension and Lyapunov exponents. These two concepts, as immutable properties of systems, are also a tool for detecting chaos in time series. A primary goal to analyze the chaotic is that this type of behavior can explain seemingly random fluctuations in financial markets and macroeconomics. But as stated, what is important is the existence of a low-dimensional chaotic system because a random process has continuous (high) dimensions while a chaotic system has more dimensions. So, one can calculate the dimensions of a series to create a process it found. According to this method, if the amplitude of the series were high, then it indicates a stochastic process; otherwise, it is a chaotic. Therefore, if the system follows a chaotic process, short-term forecasting ability would exist. However, conventional linear forecasting methods will not be practical and practical because linear models cannot correctly cover sudden movements and unlimited fluctuations in stock prices or other financial items, and nonlinear models should be used [34].

There are several instances of chaotic maps. In essence, a deterministic formula yields maps Chaos:

y(t)=ψ(y(t−1),y(t−2),...).

Due to the presence of chaotic behavior, ψ(y(t−1),y(t−2),...) is a nonlinear function. However, it should be noted that just nonlinearity is not enough to have a chaotic behavior. For example, $f(x)=x^{3}$ is nonlinear, but it does not show a chaotic feature. Chaotic is subset of nonlinear process that produces very complex and irregular results. To show chaos, we will examine two chaotic models in the following sections.

### 3.1 Chaotic logistics model

The chaotic logistics model is a dynamic model that illustrates the behavior of complex financial systems in logistics and supply chain management. In this approach, the interactions between various variables are nonlinear and lead to a chaotic behavior. One of the key characteristics of the logistics models is sensitivity to initial conditions. This means that a small change in the initial conditions can lead to drastically different outcomes. This sensitivity to initial conditions can make it difficult to predict the behavior of the system in the long term. Another important feature of this model is the presence of delays in dynamics. These delays and feedback loops can strengthen small disturbances and lead to unpredictable fluctuations in the system. Delays in the system can also introduce lags in the response of the system to changes, further complicating the behavior of the system. Also, this model can provide valuable insights into the dynamics of logistics and supply chain systems. By studying the chaotic behavior of these systems, researchers and practitioners can better understand the underlying mechanisms that drive their behavior and develop strategies to optimize their performance.

**Figure 2:**
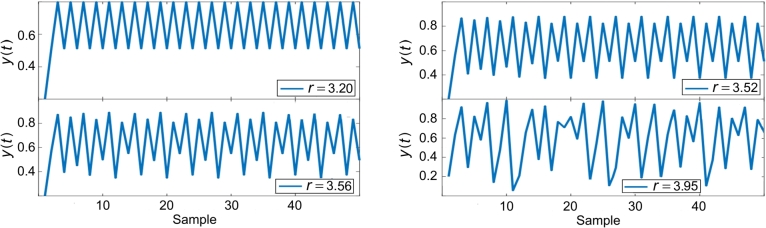
Chaotic logistics model.

We examine the most common and most straightforward chaotic system, known as logistic mapping, and includes the receiver of a one-variable, first-order nonlinear differential equation. This mapping is defined as follows:

(1) y(t+1)=r⋅y(t)(1−y(t)),

where *r* is the logistic coefficient. In Eq. (1), coefficient *r* causes the effect of butterflies. For a better understanding of the issue, a one-dimensional logistic map of y(t) changes with the assumption of r=3.95 and y(0)=0.2 is shown in Fig. 2, where y(0) represents the starting point. The changes of y(t) appear to be totally random, and no specific pattern can be considered. But when we examine the relationship of variables in successive repetitions in a two-dimensional map, the process of data changes will have a definite pattern. Assume that another specific process of change is reached.

**Figure 3:**
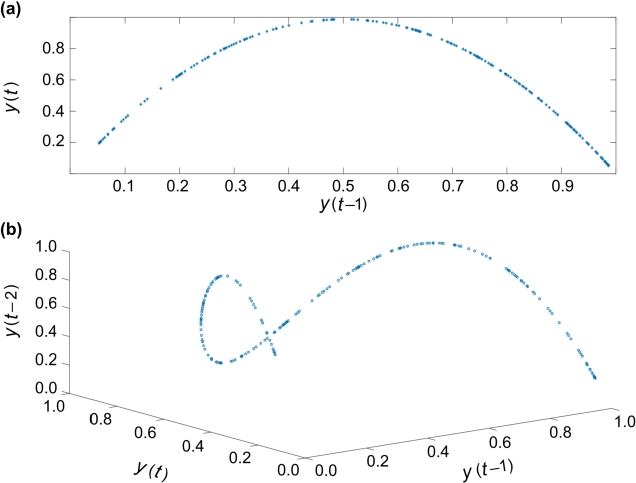
Chaotic logistics model: (a) 2D, and (b) 3D.

Sensitivity to beginning circumstances is one of a chaotic system's fundamental properties. To clarify this issue, it is better to examine the sensitivity of the logistic map in one-dimensional space for different values of *r*. In logistic mapping, by placing the initial point y(0)=0.2 and changes, *r*, the nature of the time series can be seen differently. The sensitivity of logistic mapping behavior according to different values of *r* is shown in Table 1. For some values of *r* from the intervals stated in Table 1, the one-dimensional logistic map is drawn in Fig. 2. As it is clear in the figure, a very small change in the value of *r* causes drastic changes in the results. Fig. 3(a) and 3(b) illustrate some diagrams of the two and three dimensions of the chaotic logistics model, respectively.

**Table 1:** The sensitivity of Eq. (1) according to different values of *r*.

0 < *r* < 1	y(t) tends to zero.
1 < *r* < 3	y(t) tends to (*r* − 1)/*r*.
3 < *r* < 3.45	y(t) tends to a two-period cycle.
3.45 < *r* < 3.56	y(t) tends to a four-period cycle.
3.56 < *r* < 3.57	y(t) tends to a cycle with even periods.
3.57 < *r* < 4	y(t) turns into chaotic fluctuations.
*r* > 4	y(t) tends to minus infinity.

As can be observed, when 3.57≤r≤4, the logistic mapping shows an apparently irregular behavior, named here as chaotic behavior. It is also possible to analyze the initial starting point of this sensitivity. The first batch is fifty examples of logistic map output without any changes. But in the second category, the output of sample 25 is increased by 1%. The changes obtained between the first and second category show the sensitivity to the initial conditions.

### 3.2 Henon's chaotic model

**Figure 4:**
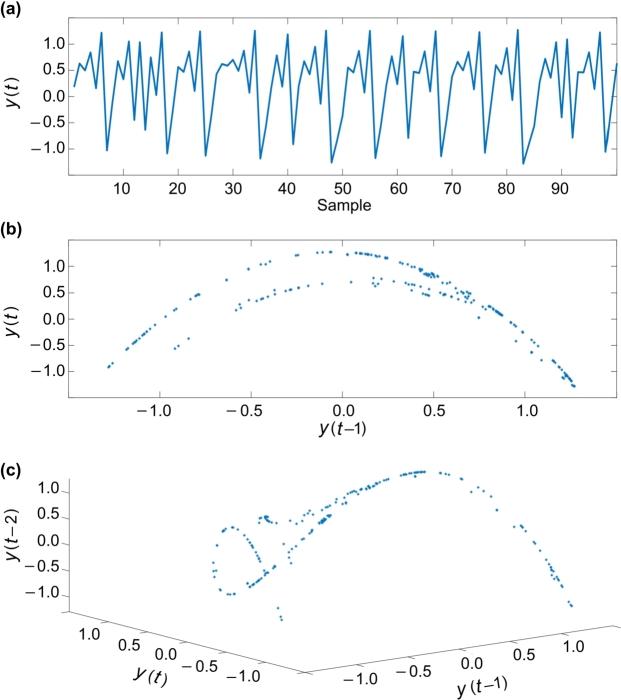
Henon's chaotic model: (a) series, (b) 2D, and (c) 3D.

Another chaotic map is Henon's model. This model has two variables, and it is nonlinear and second order. The Henon's model proposes a discrete model for chaotic modeling of financial systems. This model includes two variables. Following, the behavior of Henon's model is described in some figures. The relationships of this model are as follows:

$$\begin{array}{c} y\left(t+1\right)=1-\alpha y^{2}\left(t\right)+x\left(t\right),\\ x\left(t+1\right)=\beta x\left(t\right), \end{array}$$

which is chaotic in the area, α=1.4, β=0.3. Initial conditions of the system are as $y_{0}=0.18$, $y_{1}=0.63$. In figures, the relationship of variables in one-dimensional and two-dimensional space is displayed. The diagrams for one, two and three dimensions of Henon's chaotic model are depicted in Fig. 4(a), 4(b), and 4(c), respectively.

### 3.3 Chaos tests

Generally, two viewpoints have been put out to assess the condition of complicated time series. In the first point of view, it is looked into whether a specific or random process produced the required time series. The second viewpoint makes an effort to assess whether the time series shows chaotic or non-chaotic behavior. The first perspective's procedures are based on an examination of the system's correlation dimension. The examination of the greatest Lyapunov representation is one of the approaches used to support the second point of view, and it is briefly detailed below.

### 3.4 The concept of the Lyapunov view

Lyapunov expressions are defined as the logarithm of the absolute magnitude of the eigenvalues of the linearized dynamics of the system on the absorber and can be calculated for continuous and discrete systems. Positive Lyapunov profiles indicate divergence, whereas negative Lyapunov profiles indicate convergence (stability) (instability). The Lyapunov spectra set is employed to gauge the impact of starting circumstances on a chaotic dynamical system prior to the development of the chaos theory. Chaos theory merely asserts that positive and negative Lyapunov exponent values can coexist in a naturally chaotic system without contradictory definitions. Since the Lyapunov exponents are by definition independent of the initial circumstances and the absorption route has several stationary characteristics, they are an expression of the degree of dynamic complexity, and in a chaotic system, their maximum positive value serves as the upper bound of the prediction.

The primary characteristic of these series, i.e., the chaotic level of these series, assessed by Lyapunov coefficients, is given attention to determine the predictability of the time series of the financial markets. This rate describes how much altering the initial circumstances or model parameters changed the generated series from the original series. Suppose a time-varying model properly predicts how a natural system will behave. This time-varying deterministic model may be the result of $\overset{˙}{y}\left(t\right)=\psi (y\left(t\right))$ Or, in the discrete case, the answer to the equation is y(t+1)=ψ(y(t)), which *ψ* is considered derivable in this case. Still, the initial conditions Δy(0) are determined by an imprecise error. The amount of error resulting from the error of the initial conditions at time Dy(t) has behavior as $e^{\lambda t}\Delta y\left(0\right)$ in which Δ indicates the amount of the error of the initial conditions. To calculate the Lyapunov representation of the partial *m* vectors, the following vectors are used.

Y(t)=[y(t),y(t+1),...,y(t+m)].

From the vectors with a distance less than *r* in the form of

$$r\left(m;i,j\right)=\left\|Y_{i}^{m}-Y_{j}^{m}\right\|\leq r,$$

the following expression is calculated.

$$d_{n}\left(m;i,j\right)=\frac{\left\|Y_{i+n}^{m}-Y_{j+n}^{m}\right\|}{r_{0}\left(m;i,j\right)}.$$

Then the largest Lyapunov representation is calculated:

$$L_{e}\left(m,n\right)=\sum_{a\neq b}\frac{\log\left[d_{n}\left(m;a,b\right)\right]}{N\left(N-1\right)}.$$

The positive value of $L_{e}$ indicates the chaotic nature, and its negative value indicates the non-chaotic and predictable nature of the process in the long term. If $L_{e}$ takes a positive value close to zero, the chaos is weaker and medium-term forecasting is possible.

## 4 Attractor dimension test

The Attractor dimension test is a mathematical tool used to analyze and understand how complex systems evolve and behave over time. It is based on chaos theory and is commonly used in physics, engineering, and other fields that deal with the study of dynamics. The attractor dimension test measures the degree of complexity in a system by analyzing the geometric pattern formed by the system's state variables as they change over time. This helps researchers to identify and study patterns in the behavior of complex systems, such as weather patterns and the behavior of financial markets. The Attractor dimension test is a valuable tool that helps researchers to model and predict the behavior of complex systems and has numerous applications in various fields.

This test depends on one of the unique traits that distinguishes a random process from a chaotic process. The dimensions of a random process are continuous (infinite). A chaotic system, however, has much more constrained dimensions. It therefore has a set of one of the points, where the temporal route is constrained herein. Therefore, it is possible to understand the process that creates it by calculating the dimensions of a series. According to this method, if the range of the above series (usually greater than 10) indicates a process is stochastic, otherwise it would be chaotic. Dimension is defined as the lower limit of the number of independent variables necessary to describe the absorber model. The fractal dimension of absorber of chaotic processes is called a strange-attractor. The strange-attractor is a fractal geometric structure that is characterized by the asymptotic states of the chaotic system. Fractals are mathematical objects. Are the same on different scales. In the strange absorber, the trajectory covers the state of the nonlinear absorber as a density it meets every point at a distance of *ε* from the previous path, and the paths never repeat. This property causes a complex behavior, apparently random but certain. The error in estimating each absorbing point can cause the prediction system to lead another path and make it impossible to predict in the following stages. The absorber dimension is calculated by correlation integral, as follows.

Suppose that $y_{t}$, t=1,2,...,T is a time series and is defined in the m-dimensional space, that is, $Y_{t}^{M}=\left[y_{t},y_{t-1},...,y_{t+M-1}\right]$ if the real *n*-dimensional system and, m≥2n+1, then these *m*-dimensional points can create the dynamics that the system under investigation created. The spatial correlation between these m-dimensional sets is obtained by calculating the correlation integral. For future studies, the suggested method can be improved by the fuzzy approach of [35], [36], [37], [38], Cohen-Grossberg neural networks (NNs) [39], discrete-time neutral-type NNs [40], numerical modeling [41], and fuzzy fractional modeling [42], [43].

## 5 Simulations

Most of the tests related to the discovery of the chaotic process in a time series require a lot of data. The data used in this research are the data related to the currency market, which is available on a daily basis. The data used in this article are divided into two parts: synthetic and experimental data. Synthetic data are chaotic data generated with deterministic systems whose deterministic equations were explained in the previous sections. Among the most famous chaotic maps, we can refer to Logistics Map and Henon Map. The number of data used in each system to find Lyapunov expressions is 4537 observations. The experimental data of the exchange rate data of the American dollar (USD against the Iranian rial and also the British pound), (GBP, Euro), EUR, Canadian dollars (CAD and Australia), (AUD, Swiss franc), (CHF, one hundred Japanese yen), (JPY100 Swedish kronor), (SEK) Norway (NOK) and UAE dirham (AED) have been defined against the Iranian rial from 05/01/1992 to 02/03/2007. The output of the software simulations consists of two parts and is in the form of Excel, the first part represents the Lyapunov view, the first column of the Lyapunov view represents one dimension, and all the numbers obtained in that column are the Lyapunov view, which is the largest number obtained in each. The column represents the maximum Lyapunov view. The second part is the absorber dimension, the last number obtained in each column represents the dimension of the system (Henon Maps). The final results are given in the Table 2, Table 3, Table 4, Table 5.

**Table 2:** The results for x(t+1)=rx(t)(1−x(t)),x(0)=0.2,r=3.94.

J	Max	J	Max
1	0.6951	6	0.0574
2	0.4555	7	0.0310
3	0.5334	8	0.0212
4	0.2085	9	0.0115
5	0.1127	10	0.0114

**Table 3:** The results for $y\left(t+1\right)=1-\alpha y^{2}\left(t\right)+y\left(t\right),\;y\left(0\right)=0.1,\;\alpha =1.4,\;\beta =0.3$.

J	Max	J	Max
1	0.3092	6	0.0547
2	4.9317	7	0.0325
3	0.2529	8	0.0204
4	0.1786	9	0.0243
5	0.0923	10	0.0078

**Table 4:** Calculation of maximum Lyapunov views in dimension one to five.

	1	2	3	4	5
USD	1.4875	0.0707	9.6710	0.1057	0.0152
NOK	12.4671	0.3804	2.9626	0.0904	0.0402
EUR	9.8415	0.1381	0.7295	0.0540	0.0280
CAD	11.6684	1.6253	0.0762	0.1901	0.0392
CHF	12.6315	0.1607	0.8663	0.0768	0.0384
GBP	11.9020	0.1728	0.8545	0.0732	0.0371
JPY	12.3558	0.1735	0.7481	0.0698	0.0423
AUD	12.2360	0.1658	1.0844	0.0692	0.0371
SEK	11.7690	2.9805	0.0702	0.3200	0.0390
AED	6.15664	0.3772	9.3964	0.1883	0.2120

**Table 5:** Calculation of maximum Lyapunov views in dimension five to ten.

	6	7	8	9	10
USD	0.0151	0.0145	0.0147	0.0148	0.0132
NOK	0.0251	0.0160	0.0117	0.0100	0.0091
EUR	0.0169	0.0104	0.0085	0.0057	0.0045
CAD	0.0288	0.0146	0.0094	0.0106	0.0073
CHF	0.0241	0.0153	0.0188	0.0113	0.0106
GBP	0.0239	0.0145	0.0085	0.0054	0.0038
JPY	0.0230	0.0177	0.0145	0.0123	0.0082
AUD	0.0207	0.0124	0.0099	0.0068	0.0055
SEK	0.0221	0.0157	0.0099	0.0083	0.0074
AED	0.0971	0.1037	0.0378	0.0315	0.0242

The similarity reports the values of the largest view in each dimension in the experimental and synthetic studied series, which indicates the existence of a certain degree of certainty in the experimental data. Secondly, it can be seen that, except for the US dollar and the UAE dirham, in other parity rates, the increase in the hedging dimension (J) leads to the shrinking of the largest Lyapunov surface (Max), which means that the dynamics of the exchange rate of the dollar and the dirham are more sensitive than other currencies. As it can be seen in Table 5, the absorptive dimension for the two logistic and Henson maps (0.44 and 1.04) is close to zero, which shows that these models are non-random and the certainty of the model and their predictability are established. The absorption dimension obtained for the countries shows that the dynamics of the system are not very complicated and short-term forecasting can be investigated.

**Figure 5:**
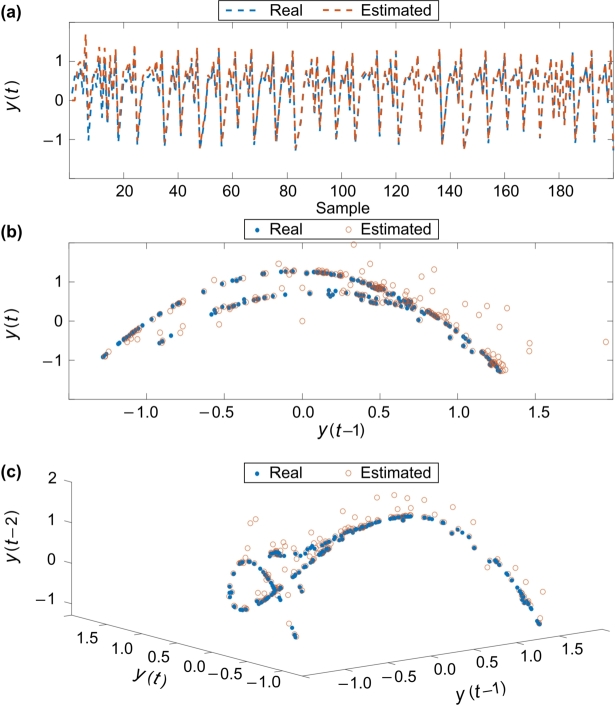
The estimated and real signals for Henon's chaotic model: (a) series, (b) 2D, and (c) 3D.

**Figure 6:**
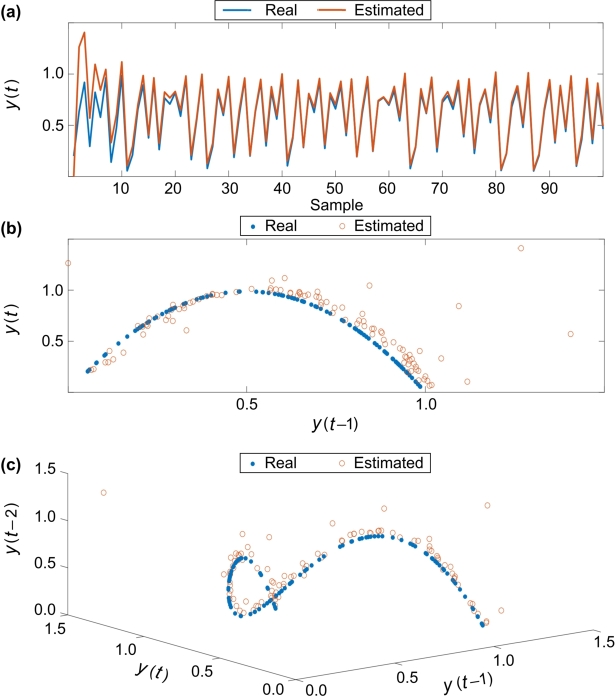
The estimated and real signals for logistics chaotic model: (a) series, (b) 2D, and (c) 3D.

To examine the ability of T3-FLS based model, the accuracy is examined on both above mentioned chaotic models. Fig. 5(a), 5(b), and 5(c) illustrate the results of series, 2D, and 3D for Henon's chaotic model, respectively. Also, the results of series, 2D, and 3D for the logistics chaotic model are depicted in Fig. 6(a), 6(b), and 6(c), respectively. As the results show, the suggested approach well models the complicated chaotic dynamics.

## 6 Conclusion

In this paper, a new T3-FLS based approach was proposed for analyzing financial chaotic systems. An important reason for examining chaotic behavior here was that this type of behavior could potentially explain the seemingly random fluctuations of financial markets and macroeconomics. The attracting dimension tests and the largest Lyapunov exponents were used to indicate the chaos and the complexity level. The suggested approach was used for modeling two basic chaotic models, namely, Logistics and Henon systems, and the results showed good modeling accuracy. In addition, the suggested approach was applied to discover the chaotic behavior in the time series of exchange rate parity in the financial markets. The suggested T3-FLS showed excellent accuracy in modeling. However, to improve the robustness and increase the interpretability, the rules of T3-FLS can be optimized.

## CRediT authorship contribution statement

**Shu-Rong Yan:** Writing – original draft, Methodology, Formal analysis, Conceptualization. **Ardashir Mohammadzadeh:** Writing – review & editing, Supervision, Conceptualization. **Ebrahim Ghaderpour:** Writing – review & editing, Visualization, Supervision, Funding acquisition, Conceptualization.

## Declaration of Competing Interest

The authors declare that they have no known competing financial interests or personal relationships that could have appeared to influence the work reported in this paper.

## Data Availability

The data employed in this research can be made available upon request.

## References

[1] Stutzer M.J. (1980). Chaotic dynamics and bifurcation in a macro model. J. Econ. Dyn. Control.

[2] Barnett W., Chen P. (1988). Deterministic chaos and fractal attractors as tools for nonparametric dynamical econometric inference: with an application to the divisia monetary aggregates. Math. Comput. Model..

[3] Shintani M., Linton O. (2003). Is there chaos in the world economy? A nonparametric test using consistent standard errors. Int. Econ. Rev..

[4] Scarlat E., Stan C., Cristescu C. (2007). Chaotic features in Romanian transition economy as reflected onto the currency exchange rate. Chaos Solitons Fractals.

[5] Lee-Chua Q.N. (1999). Dance of chaos: the application of chaos theory in the Philippine foreign exchange market. Philipp. Stud..

[6] Tian M.-W., Wang L., Yan S.-R., Tian X.-X., Liu Z.-Q., Rodrigues J.J.P. (2019). Research on financial technology innovation and application based on 5g network. IEEE Access.

[7] Cooley T.F., Quadrini V. (2001). Financial markets and firm dynamics. Am. Econ. Rev..

[8] Michael F., Johnson M. (2003). Financial market dynamics. Phys. A, Stat. Mech. Appl..

[9] Dieci R., He X.-Z., Hommes C. (2014).

[10] Diouf M., Sene N. (2020). Analysis of the financial chaotic model with the fractional derivative operator. Complexity.

[11] Liping C., Khan M.A., Atangana A., Kumar S. (2021). A new financial chaotic model in Atangana-Baleanu stochastic fractional differential equations. Alex. Eng. J..

[12] Atangana A., Bonyah E., Elsadany A. (2020). A fractional order optimal 4d chaotic financial model with Mittag-Leffler law. Chin. J. Phys..

[13] Farman M., Akgül A., Baleanu D., Imtiaz S., Ahmad A. (2020). Analysis of fractional order chaotic financial model with minimum interest rate impact. Fractal Fract..

[14] Xu C., Liao M., Li P., Xiao Q., Yuan S. (2019). Control strategy for a fractional-order chaotic financial model. Complexity.

[15] Ma Y., Li W. (2020). Application and research of fractional differential equations in dynamic analysis of supply chain financial chaotic system. Chaos Solitons Fractals.

[16] Lu X. (2020). A financial chaotic system control method based on intermittent controller. Math. Probl. Eng..

[17] Anh V., Inoue A. (2005). Financial markets with memory I: dynamic models. Stoch. Anal. Appl..

[18] Wen C., Yang J. (2019). Complexity evolution of chaotic financial systems based on fractional calculus. Chaos Solitons Fractals.

[19] Zhang X.-D., Liu X.-D., Zheng Y., Liu C. (2013). Chaotic dynamic behavior analysis and control for a financial risk system. Chin. Phys. B.

[20] Litimi H., BenSaida A., Belkacem L., Abdallah O. (2018). Chaotic behavior in financial market volatility. J. Risk.

[21] Liu Z., Mohammadzadeh A., Turabieh H., Mafarja M., Band S.S., Mosavi A. (2021). A new online learned interval type-3 fuzzy control system for solar energy management systems. IEEE Access.

[22] Cao Y., Raise A., Mohammadzadeh A., Rathinasamy S., Band S.S., Mosavi A. (2021). Deep learned recurrent type-3 fuzzy system: application for renewable energy modeling/prediction. Energy Rep..

[23] Vafaie R.H., Mohammadzadeh A., Piran M. (2021). A new type-3 fuzzy predictive controller for mems gyroscopes. Nonlinear Dyn..

[24] Wang J.-h., Tavoosi J., Mohammadzadeh A., Mobayen S., Asad J.H., Assawinchaichote W., Vu M.T., Skruch P. (2021). Non-singleton type-3 fuzzy approach for flowmeter fault detection: experimental study in a gas industry. Sensors.

[25] Castillo O., Castro J.R., Melin P. (2022). Interval type-3 fuzzy control for automated tuning of image quality in televisions. Axioms.

[26] Tian M.-W., Bouteraa Y., Alattas K.A., Yan S.-R., Alanazi A.K., Mohammadzadeh A., Mobayen S. (2022). A type-3 fuzzy approach for stabilization and synchronization of chaotic systems: applicable for financial and physical chaotic systems. Complexity.

[27] Xu A., Tian M.-W., Kausar N., Mohammadzadeh A., Pamucar D., Ozbilge E. (2023). Optimal type-3 fuzzy control and analysis of complicated financial systems. J. Intell. Fuzzy Syst..

[28] Castillo O., Castro J.R., Melin P. (2022). Interval type-3 fuzzy aggregation of neural networks for multiple time series prediction: the case of financial forecasting. Axioms.

[29] Tarafdar A., Majumder P., Bera U.K. (2023). An advanced learned type-3 fuzzy logic-based hybrid system to optimize inventory cost for a new business policy. Proc. Natl. Acad. Sci. India Sect. A Phys. Sci..

[30] Tian M.-W., Yan S.-R., Liu J., Alattas K.A., Mohammadzadeh A. (2022). A new type-3 fuzzy logic approach for chaotic systems: robust learning algorithm. Mathematics.

[31] Wu L., Huang H., Wang M., Alattas K.A., Mohammadzadeh A., Ghaderpour E. (2023). Optimal control of non-holonomic robotic systems based on type-3 fuzzy model. IEEE Access.

[32] Bie H., Li P., Chen F., Ghaderpour E. (2023). An observer-based type-3 fuzzy control for non-holonomic wheeled robots. Symmetry.

[33] Mohammadzadeh A., Sabzalian M.H., Zhang W. (2019). An interval type-3 fuzzy system and a new online fractional-order learning algorithm: theory and practice. IEEE Trans. Fuzzy Syst..

[34] Chen F., Qiu X., Alattas K., Mohammadzadeh A., Ghaderpour E. (2022). A new fuzzy robust control for linear parameter-varying systems. Mathematics.

[35] Das A.K., Granados C. (2022). Fp-intuitionistic multi fuzzy n-soft set and its induced fp-hesitant n soft set in decision-making. Decis. Mak. Appl. Manag. Eng..

[36] Ashraf A., Ullah K., Hussain A., Bari M. (2022). Interval-valued picture fuzzy Maclaurin symmetric mean operator with application in multiple attribute decision-making. Rep. Mech. Eng..

[37] Mollaei M. (2023). Fuzzy metric topology space and manifold. J. Fuzzy Ext. Appl..

[38] Adak A., Kumar D. (2023). Spherical distance measurement method for solving mcdm problems under Pythagorean fuzzy environment. J. Fuzzy Ext. Appl..

[39] Liu Y., Liu W., Obaid M.A., Abbas I.A. (2016). Exponential stability of Markovian jumping Cohen–Grossberg neural networks with mixed mode-dependent time-delays. Neurocomputing.

[40] Du B., Liu Y., Abbas I.A. (2016). Existence and asymptotic behavior results of periodic solution for discrete-time neutral-type neural networks. J. Franklin Inst..

[41] Abouelmagd E.I., Awad M., Elzayat E., Abbas I.A. (2014). Reduction the secular solution to periodic solution in the generalized restricted three-body problem. Astrophys. Space Sci..

[42] Abu Arqub O., Singh J., Alhodaly M. (2023). Adaptation of kernel functions-based approach with Atangana–Baleanu–Caputo distributed order derivative for solutions of fuzzy fractional Volterra and Fredholm integrodifferential equations. Math. Methods Appl. Sci..

[43] Abu Arqub O., Singh J., Maayah B., Alhodaly M. (2023). Reproducing kernel approach for numerical solutions of fuzzy fractional initial value problems under the Mittag–Leffler kernel differential operator. Math. Methods Appl. Sci..

